# Inhibition of transcription factor nuclear factor-**κ**B by a mutant inhibitor-**κ**B**α** attenuates resistance of human head and neck squamous cell carcinoma to TNF-**α** caspase-mediated cell death

**DOI:** 10.1054/bjoc.2000.1423

**Published:** 2000-10-26

**Authors:** D C Duffey, C V Crowl-Bancroft, Z Chen, F G Ondrey, M Nejad-Sattari, G Dong, C Van Waes

**Affiliations:** Tumor Biology Section, Head and Neck Surgery Branch, National Institute on Deafness and Other Communication Disorders, National Institutes of Health, Bethesda, MD, USA; Howard Hughes Medical Institute, Bethesda, MD, USA

**Keywords:** transcription factors, squamous cell carcinoma, NF-κB, cytokins, TNF-α

## Abstract

Tumour necrosis factor-α (TNF-α) is a cytokine that can induce cell death of different cancers via a cellular cascade of proteases, the caspases. However, TNF-α has been detected in tumour and serum of patients with head and neck squamous cell carcinoma (HNSCC), and tumour cell lines derived from this environment often exhibit resistance to TNF-α-induced cell death. Cell death mediated by TNF-α and caspases may be inhibited by cytoprotective genes regulated by transcription factor nuclear factor-κB (NF-κB). We recently showed that NF-κB is constitutively activated in HNSCC, and that inhibition of NF-κB by expression of a nondegradable mutant inhibitor of NF-κB, IκBαM, markedly decreased survival and growth of HNSCC cells in vivo. In the present study, we examined the TNF-α sensitivity and response of HNSCC with constitutively active NF-κB, and of HNSCC cells in which NF-κB is inhibited by stable expression of a dominant negative mutant inhibitor, IκBαM. Human lines UM-SCC-9, 11B and 38, previously shown to exhibit constitutive activation of NF-κB, were found to be highly resistant to growth inhibition by up to 10^4^U/ml of TNF-α in 5 day MTT assay. These TNF-α resistant HNSCC lines expressed TNF receptor I, and exhibited constitutive and TNF-α-inducible activation of NF-κB as demonstrated by nuclear localization of NF-κB p65 by immunohistochemistry. UM-SCC-9 I11 cells which stably expressed an inhibitor of NF-κB, IκBαm, were susceptible to TNF-α-induced growth inhibition. DNA cell cycle analysis revealed that TNF-α induced growth inhibition was associated with accumulation of cells with sub-G0/G1 DNA content. Cell death was demonstrated by trypan blue staining, and was blocked by caspase inhibitor. We conclude that HNSCC that exhibit constitutive and TNF-α-inducible activation of transcription factor NF-κB are resistant to TNF-α, and that inhibition of NF-κB sensitizes HNSCC to TNF-α caspase-mediated cytotoxicity. The demonstration of the role of activation of NF-κB in resistance of HNSCC to TNF-α may be helpful in the identification of potential targets for pharmacological, molecular and immune therapy of HNSCC. © 2000 Cancer Research Campaign


British Journal of Cancer (2000) **83**(10), 1367–1374
© 2000 Cancer Research Campaign
doi: 10.1054/ bjoc.2000.1423, available online at http://www.idealibrary.com on IDEAL®

### Inhibition of transcription factor nuclear factor-κB by a mutant inhibitor-κBα attenuates resistance of human head and neck squamous cell carcinoma to TNF-α caspase-mediated cell death

**DC Duffey[1], CV Crowl-Bancroft[1], Z Chen[1], FG Ondrey[1], M Nejad-Sattari[2], G Dong[1] and C Van Waes[1]***

[1]Tumor Biology Section, Head and Neck Surgery Branch, National Institute on Deafness and Other Communication Disorders, National Institutes of Health, Bethesda, MD, USA; [2]Howard Hughes Medical Institute, Bethesda, MD, USA

**Summary** Tumour necrosis factor-α (TNF-α) is a cytokine that can induce cell death of different cancers via a cellular cascade of proteases, the caspases. However, TNF-α has been detected in tumour and serum of patients with head and neck squamous cell carcinoma (HNSCC), and tumour cell lines derived from this environment often exhibit resistance to TNF-α-induced cell death. Cell death mediated by TNF-α and caspases may be inhibited by cytoprotective genes regulated by transcription factor nuclear factor-κB (NF-κB). We recently showed that NF-κB is constitutively activated in HNSCC, and that inhibition of NF-κB by expression of a nondegradable mutant inhibitor of NF-κB, IκBαM, markedly decreased survival and growth of HNSCC cells in vivo. In the present study, we examined the TNF-α sensitivity and response of HNSCC with constitutively active NF-κB, and of HNSCC cells in which NF-κB is inhibited by stable expression of a dominant negative mutant inhibitor, IκBαM. Human lines UM-SCC-9, 11B and 38, previously shown to exhibit constitutive activation of NF-κB, were found to be highly resistant to growth inhibition by up to $10^4$ U/ml of TNF-α in 5 day MTT assay. These TNF-α resistant HNSCC lines expressed TNF receptor I, and exhibited constitutive and TNF-α-inducible activation of NF-κB as demonstrated by nuclear localization of NF-κB p65 by immunohistochemistry. UM-SCC-9 I11 cells which stably expressed an inhibitor of NF-κB, IκBαm, were susceptible to TNF-α-induced growth inhibition. DNA cell cycle analysis revealed that TNF-α induced growth inhibition was associated with accumulation of cells with sub-G0/G1 DNA content. Cell death was demonstrated by trypan blue staining, and was blocked by caspase inhibitor. We conclude that HNSCC that exhibit constitutive and TNF-α-inducible activation of transcription factor NF-κB are resistant to TNF-α, and that inhibition of NF-κB sensitizes HNSCC to TNF-α caspase-mediated cytotoxicity. The demonstration of the role of activation of NF-κB in resistance of HNSCC to TNF-α may be helpful in the identification of potential targets for pharmacological, molecular and immune therapy of HNSCC. © 2000 Cancer Research Campaign

**Keywords**: transcription factors; squamous cell carcinoma; NF-κB; cytokins; TNF-α

Tumour necrosis factor-alpha (TNF-α) is a cytokine which was initially reported to have cytocidal activity against a variety of normal and neoplastic cells (Carswell et al, 1975; Haranaka and Satomi, 1981; Sugarman et al, 1985; Fransen et al, 1986). TNF-α has been shown to induce cell death of tumours via apoptosis or necrosis (Schmid et al, 1986; Dealtry et al, 1987; Larrick and Wright, 1990; Laster et al, 1998). TNF-α can induce apoptosis of some HNSCC cell lines at concentrations at or above $10^4$ U/ml (Briskin et al, 1996), but most human HNSCC have been reported to be relatively resistant to TNF-α (Gapany et al, 1990, Schuger et al, 1990; Sacchi et al, 1991, Monchimatsu et al, 1993, Briskin et al, 1996). Development of resistance to TNF-α has been shown to occur with tumour progression in murine fibrosarcomas through exposure of tumour cells to TNF-α produced by host responses, and selection of TNF-α resistant tumour cells (Urban et al, 1983, 1986). TNF-α expression has been detected in tumour and serum of patients with HNSCC, indicating that TNF-α resistant HNSCC may also develop in the presence of endogenous TNF-α (Parks et al, 1994; Soylu et al, 1994; Younes et al, 1996; Knerer et al, 1996; Kurokawa et al, 1998). Thus, these tumours may be resistant to concentrations of TNF-α produced endogenously or administered exogenously (Fraker et al, 1995; Olieman et al, 1999). The mechanism of increased resistance of HNSCC to TNF-α has not been previously defined.

TNF-α induces cell death through activation of TNF Receptor I (Tartaglia and Goeddel, 1992) and a cascade of death gene products, including caspases (Wallach et al, 1999). Lack of TNF receptor expression has been proposed as a possible basis for TNF-α resistance of HNSCC (Younes et al, 1996), but other investigators have found that TNF-α resistant HNSCC may retain expression of TNF receptors (von Biberstein et al, 1995). Alternatively, resistance to TNF-α could involve mechanisms which promote cell survival. We recently reported that survival of HNSCC cells is promoted by constitutive activation of nuclear factor-κB (NF-κB) (Duffey et al, 1999), a transcription factor which has been reported to induce expression of a variety of

*Received 20 March 2000*
*Revised 12 June 2000*
*Accepted 14 June 2000*

*Correspondence to:* C Van Waes proteins that can inhibit cell death (Beg and Baltimore et al, 1996; Wang et al, 1996; Van Antwerp et al, 1996; Mayo et al, 1997; Sun and Carpenter, 1998; Wang et al, 1998; Zong et al, 1999). We showed that several human HNSCC cell lines in the University of Michigan (UM-SCC) series exhibit constitutive activation of NF-κB and NF-κB inducible cytokine genes (Duffey et al, 1999; Ondrey et al, 1999). We also demonstrated that an increase in constitutive activation of NF-κB and expression of NF-κB inducible cytokines occurs with metastatic tumour progression in a murine model of squamous cell carcinoma (Dong et al, 1999). We noted that TNF-α induced NF-κB and NF-κB-inducible cytokine production in these human UM-SCC and murine SCC cell lines without evidence of significant cell toxicity or death. These observations suggest the hypothesis that acquisition of TNF-α resistance by HNSCC may result from selection of cancer cells in which NF-κB and cytoprotective responses can be activated.

Activation of NF-κB has been shown to involve signal-induced phosphorylation and degradation of inhibitor κB (IκB) proteins, which release NF-κB for nuclear translocation (Brockman et al, 1995; Brown et al, 1995; Traeckner et al, 1995; Verma et al, 1995), and for binding to the promoter sites of target genes. Studies in these laboratories have shown that mutations in the serine phosphorylation sites at S32 and/or S36 of IκBα can inhibit the signal-dependent activation of NF-κB by a variety of stimuli. Such phosphorylation mutants can therefore exert a dominant negative effect, preventing the activation of NF-κB dependent genes. By expression of a dominant negative IκBα mutant, NF-κB has been shown to be important in activation of genes necessary for survival and protection of cells from injury by a variety of cytotoxic stimuli, including cytokine TNF-α, chemo- and radiation therapy (Beg and Baltimore et al, 1996; Van Antwerp et al, 1996; Wang et al, 1996). In these studies, decreased resistance of cells to TNF-α-induced cell death could be demonstrated following cytoplasmic inactivation of NF-κB by expression of an inhibitor-κB (IκB) phosphorylation mutant which is unable to undergo TNF-α-induced phosphorylation and degradation. We recently reported that inactivation of NF-κB by expression of an IκB phosphorylation mutant inhibits survival and in vivo growth of human UM-SCC cell lines (Duffey et al, 1999).

In the present study, we determined the effects of TNF-α treatment on UM-SCC cell lines which exhibit constitutive activation of NF-κB, and asked whether inhibition of NF-κB activation by stable expression of a dominant negative inhibitor-κB could enhance sensitivity to TNF-induced cytotoxicity. We provide evidence that HNSCC that exhibit constitutive and TNF-α-inducible activation of transcription factor NF-κB are resistant to TNF-α, and that inhibition of NF-κB activation by the expression of a phosphorylation mutant of inhibitor-κBα (IκBαM) sensitizes a UM-SCC cell line to TNF-α-mediated cell death. This TNF-α induced cytotoxicity was blocked by caspase inhibitor. We conclude that HNSCC cell line that exhibit constitutive and TNF-α-inducible activation of transcription factor NF-κB are resistant to TNF-α, and that inhibition of NF-κB sensitizes HNSCC to TNF-α caspase-mediated cytotoxicity.

### MATERIALS AND METHODS

### Cell culture

Human squamous cell carcinoma cell lines were derived from advanced stage head and neck cancer patients at the University of Michigan and were a generous gift of Thomas Carey, Ph.D. Squamous carcinoma cell lines UMSCC-9, -11B, and -38 cells used in the present study were previously described (Duffey et al, 1999; Ondrey et al, 1999). These lines were cultured at 37°C, 5% $CO_2$ as adherant monolayer cultures in Minimum Essential Medium (Gibco/BRL, Gaithersburg, MD) with 10% heat-inactivated fetal calf serum (Gibco/BRL) containing 2 mM L-glutamine, and penicillin (50 μg/ml), streptomycin (50 μg/ml). Log-phase cells were routinely passaged weekly after trypsinization.

### Cell proliferation assay

Cell proliferation was quantified using an MTT-based colorimetric assay (Cell Proliferation Kit I, Boehringer Mannheim, Mannheim, Germany). HNSCC cells were plated in flat-bottomed 96-well plates at a density of $5 \times 10^3$ cells/well and allowed to adhere overnight at 37°C. Addition of control medium or medium with TNF-α was followed by incubation at 37°C for 1–5 days. The MTT assay was conducted at 1, 3 and 5 days following stimulation according to manufacturer's protocol (Boehringer Mannheim, Indianapolis, IN). At endpoint intervals, 100 μl of medium was removed and 10 μl of dimethylthiazol-diphenyl tetrazolium bromide (MTT) labelling reagent was added and the plate was incubated for 4 hours at 37°C as per the manufacturer's recommendations. After a 4 hour incubation, cells were solubilized by adding 100 μl of 10% SDS in 0.01 M HCl as per the manufacturer's instructions. Overnight incubation at 37°C was then followed by optic densitometry reading at 570 nm with a microplate reader (Biotek 311, Biotek Systems, Winooski, VT). All readings were done in quadruplicate.

### RNAse protection assay

Total RNA from UM-SCC-9, 11B and 38 was harvested with Trizol reagent (Gibco BRL Life Technology, Inc, Gaithersburg, MD). 10 μg of RNA from each sample was hybridized with $^{32}$P-labelled RNA probes specific for TNFRI and II made from commercially available templates, which included probes for L32 and GAPDH as loading controls (hCR-4, #45374P, Pharmingen, San Diego, CA). The hybridized products were digested with RNAse. 15 μg total RNA was loaded per lane and the protected RNA probes were separated by sequencing gel electrophoresis which was exposed to X-ray film. The films were scanned and density of TNFRI and TNFRII was normalized to GADPH using NIH IMAGE software, v1.62, and reported as a ratio.

### Immunohistochemical staining

Immunohistochemical analysis was performed using anti-TNF RI and anti-TNF RII, and anti-p65 antibody which recognizes the nuclear localization sequence of the activated form of NF-κB p65 using a modification of the protocol of Kaltschmidt et al (1995). UM-SCC-9, -11B and 38 cells were plated at a density of $10^4$ cells and incubated at 37°C for 2–3 days to roughly 50% confluency on 8-well chamber slides (Lab-Tek, Naperville, IL). The slides with attached cells were fixed with 3.7% formalin in PBS for 5 minutes, washed with PBS, and then permeabilized with 0.2% Triton X-100 in PBS for 10 minutes. After washing, the slides were blocked with 10% goat serum for 30 minutes, and goat anti-TNF RI or anti-TNF RII antibody (Santa Cruz Biotechnology, Santa Cruz, CA)

 © 2000 Cancer Research Campaign was added directly into the blocking serum on the slides at a 1:2000 dilution for 1 hour. Isotype controls were purified goat IgG at 1:2000 dilution (Cappel, West Chester, PA) that corresponded to an equal concentration of primary antibody. After washing, secondary anti-goat antibody and biotin-avidin conjugates from Vectastain Elite ABC kit and chromogen diaminobenzidine tetrahydrochloride (Vector Lab, Inc., Burlingame, CA) were used for colour development following the manufacturer's instructions.

### Transfection of UM-SCC-9 cells with IκBαM and control vector

The cDNA plasmid pCMX IκBαM contains a mutation at S36 of the $NH_2$ terminus and a COOH-terminal PEST sequence mutation, and was a generous gift from Dr Inder M Verma, Salk Institute, La Jolla, CA (Van Antwerp et al, 1996). The plasmid containing the neomycin (neo) resistance gene used is described by Brown et al (1995). The method of transfection and isolation of UM-SCC-9 cells was previously described (Duffey et al, 1999). UM-SCC-9 I11 cells expressed IκBαM most abundantly and UM-SCC-9 C11 cells transfected with vector control alone were expanded and used for the present studies. We recently showed that the difficulty in obtaining stable transformants of UM-SCC 11B and 38 cell lines is due to decreased survival of cells transfected with IκBα (Duffey et al, 1999).

### Cell viability by DNA cytofluorometry and trypan blue exclusion

Cells were collected for DNA cell cycle analysis and stained with propidium iodide using the Cycle TEST PLUS DNA Reagent Kit according to manufacturer's instructions (Becton Dickinson, San Jose, CA). The stained cells were analysed using a FACScan flow cytofluorometer and compared for DNA content following calibration with diploid DNA QC particles, using CELLQuest software (Becton Dickinson, Mountain View, CA). Statistical analyses were performed by ModFit LT software (Verity Software House, Topsham, ME).

Cell viability was quantified by trypan blue exclusion. Cells were plated at $5 \times 10^3$ cells/well in each well of a 96-well plate. UM-SCC-9 I11 and UM-SCC-9 C11 control cells in monolayer cultures were treated with TNF-α as described, and adherent and nonadherent cells were collected in suspension following trypsin-EDTA treatment. For the caspase inhibition study, UM-SCC9 I-11 cells were incubated overnight, pre-incubated for 60 minutes with 0, 1, 10, and 25 μM Caspase Inhibitor I (Z-VAD-FMK) (Calbiochem, La Jolla, CA), and 1000 U/mL TNF-α was added. Cells were centrifuged at 1200 rpm for 5 minutes at room temperature. The cell pellet was resuspended in MEM complete medium. An aliquot was mixed with an equal volume of 1.0% trypan blue, and cell concentration and viability were determined using a haemacytometer.

### RESULTS

### UM-SCC-9, 11B and 38 cell lines are resistant to TNF-α induced cytotoxicity and express TNFR I

To determine the sensitivity of a panel of HNSCC lines to TNF-α, we cultured UM-SCC-9, 11B and 38 cell lines with 100, 1000 and $10^4$ U/ml of TNF-α or control media, and compared the proliferation of cells during a 5-day MTT assay (Fig. 1). TNF-α showed no appreciable inhibitory effect upon the proliferation of cells during the first 3 days, and only a small inhibition of growth of UM-SCC-9 and 38 cells was detected by day 5. Although the inhibition of growth following treatment at higher concentrations was statistically significant, cells continued to grow in the presence of TNF-α, and no difference in density larger than 30% was detected by day 5. The TNF-α used was functional, since TNF-α at the same concentrations completely inhibited proliferation of primary keratinocytes (not shown). Since it has been reported that resistance of HNSCC to TNF-α-induced cytotoxicity may be due to loss of expression of TNF receptor (Younes et al 1996), we examined the expression of TNF receptor I and II mRNA and protein expression, as determined by RNAse protection assay and immunohistochemical analysis. Table 1 shows that all three UM-SCC cell lines expressed quantitatively similar ratios of TNFR I mRNA when normalized to GADPH by densitometric analysis. A similar pattern of protein staining was detected in all 3 cell lines by immunohistochemistry. No TNFR II was detected in UM-SCC cells by either method, while control A549 cells were positive for both receptors by immunohistochemistry. Thus, the TNF-α resistant UM-SCC cell lines examined in the present study retain expression of TNFR I, the TNF receptor associated with TNF-inducible cell cytotoxicity (Tartaglia and Goeddel, 1992).

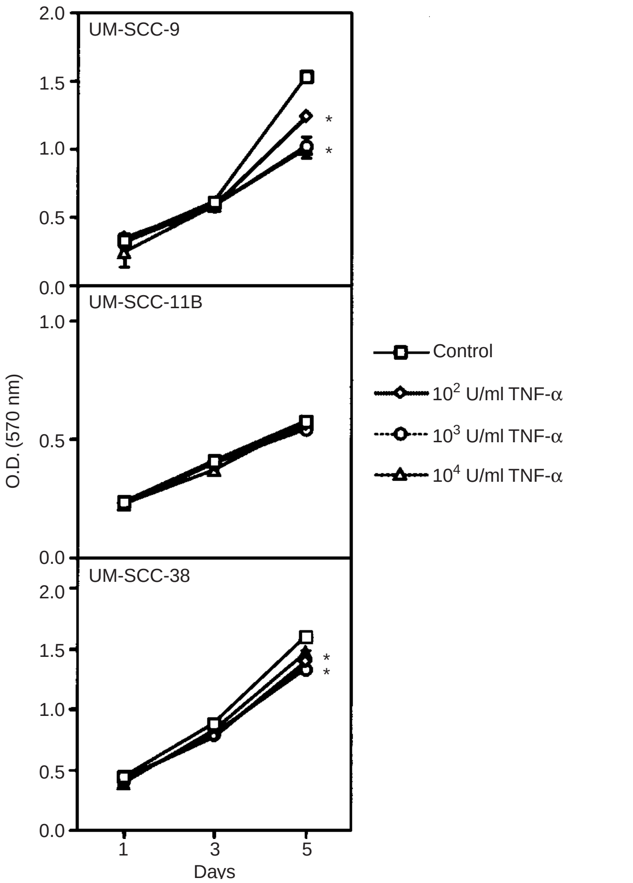

**Figure 1** Effect of TNF-α upon growth of UM-SCC cell lines in MTT assay. UM-SCC-9, 11B and 38 cells were cultured in 96 well plates in the presence of 0 to $10^4$ U/ml TNF-α, and growth on day 1, 3 and 5 was compared by MTT assay, as described in Methods. The OD 570 nm +/– SEM is shown. *Denotes significant difference by Student's *t* test at $P < 0.05$

© 2000 Cancer Research Campaign

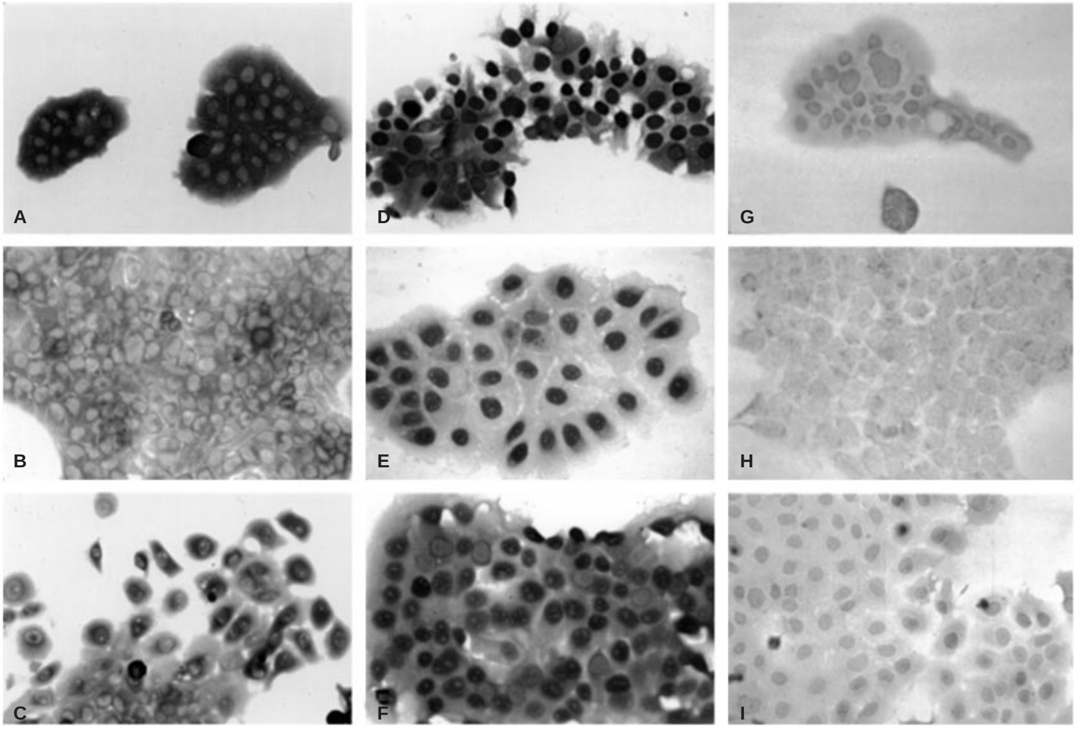

**Figure 2** Immunolocalization of activated NF-κB p65 in untreated and TNF-α treated UM-SCC cell lines. UM-SCC-38, 9, and 11B cell lines stained with anti-p65 specific for the nuclear localization sequence in the absence of TNF-α (-TNF−α) show varying levels of constitutive cytoplasmic and nuclear staining, while $10^4$ U/ml TNF-α (+TNF-α) induces increased nuclear localization of p65 in all cell lines

**Table 1** Detection of TNF receptor expression in UM-SCC cells by RNAse protection assay and immunohistochemistry

| TNF receptor | Cell line UM-SCC-9 | UM-SCC-11B | UM-SCC-38 | A549 |
|---|---|---|---|---|
| TNF RI | | | | |
| RNA | 0.82 | 0.80 | 0.78 | ND |
| Protein | + | + | + | + |
| TNF RII | | | | |
| RNA | – | – | – | ND |
| Protein | – | – | – | + |

TNFR I and II mRNA expression was assayed by RNAse protection, and the ratio of TNFR to GADPH mRNA is reported, as described in Methods. TNFR I and II protein was assayed by immunohistochemistry using anti-TNF RI and TNF RII antibodies. UM-SCC were compared with A549 cell line expression as a positive control for both TNFR I and TNFR II. ND, not done

### TNF-α induces increased activation and nuclearl localization of NF-κB in UM-SCC cell lines

Resistance to TNF-α induced cell death has been associated with activation of NF-κB (Beg and Baltimore, 1996; Van Antwerp et al, 1996; Wang et al, 1996). We previously showed that NF-κB/Rel A (p50/p65) is constitutively activated in UM-SCC-9, 11B and 38 cell lines (Ondrey et al, 1999), and may be further induced by TNF-α in UM-SCC-9 (Duffey et al, 1999). To examine whether TNF-α induces activation of NF-κB/Rel A in TNF-α resistant HNSCC cell lines, we examined the pattern of nuclear activation and localization of the NF-κB p65 subunit by immunoperoxidase staining in UM-SCC-9, 11B and 38, in the absence and presence of $10^4$ U/ml of TNF-α, using an antibody that recognizes the nuclear localization site of activated Rel A p65 (Kaltschmidt et al, 1995; Duffey et al, 1999). The left panels in Fig. 2 show the baseline staining pattern, which reveals mixed cytoplasmic and nuclear staining of p65 in UM-SCC 9 and 38 cell lines, and an apparent increase in constitutive nuclear staining in UM-SCC-11B. The apparent difference in constitutive nuclear localization between UM-SCC-11B and the other two cell lines is consistent with the relative differences in constitutive activation of NF-κB in these cell lines by EMSA and NF-κB luciferase reporter assay (Ondrey et al, 1999). Within 15 minutes of treatment with TNF-α, an increase and predominant staining of NF-κB in the nuclear and perinuclear regions was detected in all three cell lines (Fig. 2, middle panels). The staining with anti-p65 could be differentiated from background detected with an isotype control (Fig. 2, right panels). We confirmed that TNF-α induced p50/p65 DNA binding activity in the cell lines by electromobility shift assay (Ondrey et al, 1999; D Duffey, data not shown). Thus, TNF-α induces activation of the NF-κB signal pathway in HNSCC cell lines that are resistant to TNF-α.

### TNF-α induces cell death in UM-SCC-9 111 cells expressing a dominant negative mutant Inhibitor-κB by a caspase-dependent mechanism

We recently demonstrated that expression of an inhibitor-κBα phosphorylation mutant (IκBαM) in UM-SCC-9 can inhibit both constitutive and TNF-α inducible activation of NF-κB (Duffey et al, 1999). The inhibition of NF-κB in UM-SCC-9 I11 cells was demonstrated by EMSA, NF-κB luciferase reporter activity, and by expression of NF-κB-dependent cytokine gene expression (Duffey et al,

 © 2000 Cancer Research Campaign

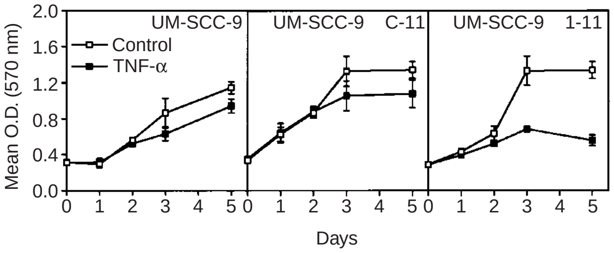

**Figure 3** Growth of UM-SCC-9, C11 and I11 cells cultured without and with TNF-α in MTT assay. UM-SCC-9 parental cells, UM-SCC-9 C11 cells transfected with vector alone, and UM-SCC-9 I11 cells transfected with IκBαM mutant were cultured in media alone or media with $10^4$ U/ml of TNF-α. Growth was compared on days 0, 1, 2, 3 and 5 of culture. The optical density shown is the average result of two independent experiments, each consisting of quadruplicate cultures. A significant inhibition in growth was observed in UM-SCC-9 I11 cells on day 3 and 5 (*P* < 0.05)

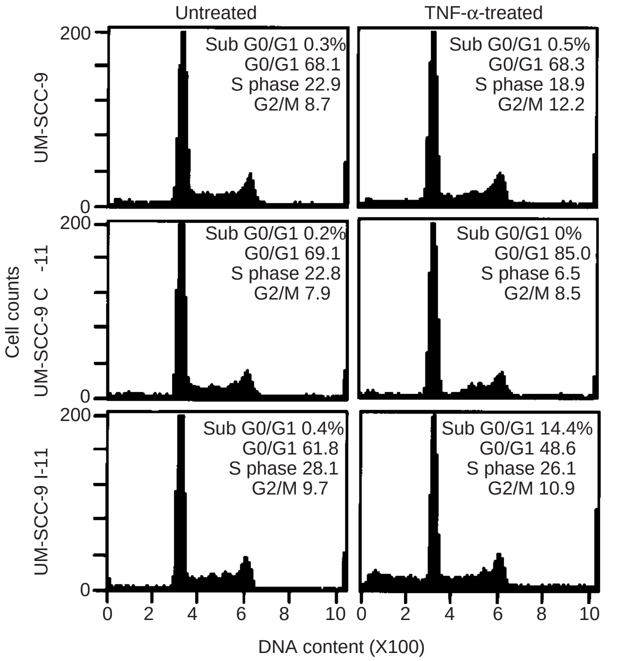

**Figure 4** DNA content analysis of UM-SCC-9, C11 and I11 cells cultured without and with TNF-α. UM-SCC-9, C-11 and I-11 cells cultured without (Control) and with TNF-α for 24 were stained with PI and analysed for DNA content by flow cytofluometry, as described in methods. The percentage fo cells in sub G1/G0, G1/G0, S and G2/M was quantified. A 14% increase in cells with sub G0/G1 DNA content was detected at 24 hours

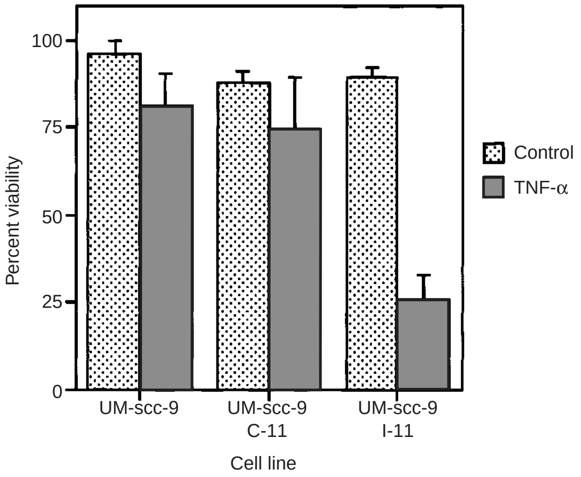

**Figure 5** Cell viability of UM-SCC-9, C11 and I11 cells cultured without and with TNF-α in trypan blue exclusion assay. The viability of cells following exposure to $10^4$ U/ml TNF-α was determined by trypan blue exclusion and staining as described in Methods. A 75% decrease in viability observed in I11 cells was significant (*P* < 0.05)

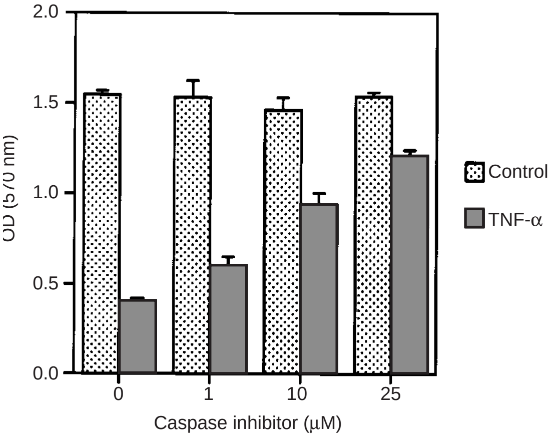

**Figure 6** TNF-α induced cell death is blocked by Caspase I inhibitor. Cell viability was quantified by trypan blue exclusion. For the caspase inhibition study, UM-SCC9 I-11 cells were pre-incubated for 60 minutes with 0, 1, 10, and 25 μM Caspase Inhibitor I (Z-VAD-FMK) and 1000 U/mL TNF-α was added

1999). To determine if inhibition of NF-κB sensitized UM-SCC-9 cells to TNF-α, we compared the TNF-α sensitivity of IκBαM transfected UM-SCC-9 I11 cells with UM-SCC-9 and control vector transfected UM-SCC-9 C11 cells in MTT assay. Figure 3 shows the average optical density from two independent MTT experiments. UM-SCC-9 I11 cells exposed to TNF-α exhibit a significant decrease in density relative to untreated UM-SCC-9 I11 or UM-SCC-9 and control vector transfected UM-SCC-9 C11 cells by day 3 of culture. An effect of TNF-α on UM-SCC-9 I11 cells is not detectable by MTT assay on day 1 and 2. The difference is observed toward the end of the exponential growth phase when untreated UM-SCC-9 I11 cells reach maximal density, and is sustained without further increase in the difference for up to 5 days of culture.

To examine if the decrease in cell density of IκBαM transfected cells treated with TNF-α is associated with evidence of cell cycle block or sub-G0/G1 DNA fragmentation prior to detection of differences in density, the DNA staining profile of IκBαM transfected cells was determined by flow cytofluorometry 24 hours following treatment with TNF-α. Figure 4 shows a comparison of propidium iodide DNA staining in UM-SCC-9, control vector, and IκBαM transfected cells following TNF-α treatment. A 14% increase in sub-G0/G1 DNA content was observed in IκBαM expressing UM-SCC-9 I11 cells beginning 24 hours following treatment with TNF-α, while no increase in sub-G0/G1 DNA staining was observed in UM-SCC-9 and UM-SCC-9C11 cells.

To establish whether the significant decrease in cell density detected after 3 days in Figure 3 was attributable to cell death, we determined the viability of cells by trypan blue exclusion at 72 hours. Decreased viability of UM-SCC-9 I11 cells was detected, as shown by a 75% decrease in cells excluding trypan blue exclusion

*© 2000 Cancer Research Campaign*

following exposure of the cells to TNF-α for 72 hours (Fig. 5). To determine if TNF-α induced cell death following inhibition of NF-κB was attributable to a caspase mediated mechanism, we determined whether cytotoxicity could be blocked by a caspase inhibitor. Figure 6 shows that caspase inhibitor blocked TNF-α induced cytotoxicity in a dose-dependent manner. We conclude that TNF-α induces cytotototoxicity in UM-SCC-9 cells expressing a dominant negative mutant inhibitor-κB by a caspase-dependent mechanism. The blockade of caspase dependent cell death by NF-κB has been shown previously to be due to NF-κB induced expression of cytoprotective proteins, which can be blocked with cycloheximide (Beg and Baltimore, 1996). We further confirmed by microscopy that TNF-α induces morphologic cell fragmentation of all 3 UM-SCC cells lines in the presence of 10 μg/ml cycloheximide, but not TNF-α or cycloheximide alone (data not shown). These observations provide evidence that NF-κB mediated resistance of the UM-SCC cell lines to TNF-α is dependent on TNF-α inducible cytoprotective proteins.

### DISCUSSION

In the present study, we confirmed that the 3 human UM-SCC cell lines previously shown to exhibit constitutive activation of NF-κB are highly resistant to TNF-α induced cell death. Our results which demonstrate a relatively high resistance of these 3 UM-SCC cell lines to TNF-α cytotoxicity are consistent with several studies with different panels of cell lines, which showed that resistance of HNSCC to TNF-α is common (Gapany et al, 1990, Schuger et al, 1990, Sacchi et al, 1991, Monchimatsu et al, 1993, Briskin et al, 1996). The UM-SCC cell lines in the present study exhibited limited sensitivity at $10^4$ U/ml TNF-α, consistent with results obtained in another laboratory with a different panel of HNSCC cell lines (Briskin et al, 1996). TNF-α has been shown to induce a wide range of biological responses, including inflammation, cell proliferation, differentiation, tumour necrosis and apoptosis (Liu et al, 1996). Induction of responses to TNF-α is mediated through binding of TNF Receptor I or II and activation of the TNF-Receptor-associated protein 1 and 2 (TRAF) pathways (Tartaglia and Goeddel et al, 1992; Wallach et al, 1999). Previous investigators have attributed a lack of TNF-α sensitivity of HNSCC to a lack of TNF receptor expression (Younes et al, 1996). We have demonstrated that the UM-SCC cell lines examined in this and our previous studies exhibit resistance to TNF-α induced cell death, while retaining expression of TNFR I. We have shown that these HNSCC retain TNF-α responsiveness, as demonstrated by TNF-α inducible activation of transcription factor NF-κB/RelA (Fig. 1; Dong et al, 1999; Duffey et al, 1999). In UM-SCC-9 cells in which we obtained stable expression of a mutant IκBα (IκBαM) and inactivation of NF-κB (Duffey et al, 1999), TNF-α inhibited growth and induced an increase in cell death relative to that observed in UM-SCC-9 cells or cells transfected with vector lacking the insert. We obtained evidence confirming that the TNF-α induced cell death observed was dependent on the caspase pathway, and that TNF-α resistance of HNSCC is dependent upon inducible expression of protective proteins, as previously reported.

In previous studies, we noted that TNF-α treatment of human and murine SCC cell lines induced NF-κB and NF-κB dependent cytokine production (Dong et al, 1999; Duffey et al, 1999), without evidence of significant cytotoxicity or cell death. We reported recently that inactivation of NF-κB by expression of an inhibitor-κB (IκB) phosphorylation mutant in human HNSCC cells can inhibit survival in vitro and growth in vivo (Duffey et al, 1999). We encountered difficulty in obtaining other HNSCC lines which stably expressed the dominant negative IκBα phosphorylation site mutant, suggesting that expression of the mutant IκBα could severely affect survival of transfected UM-SCC cells. We confirmed that when 3 UM-SCC cell lines were co-transfected with a Lac-Z reporter in the presence of excess vector containing a human IκBα phosphorylation mutant or control vector, transfection of mutant IκBα markedly reduced the survival of β-galactosidase staining cells by 70–90% in cultures within 72 hours (Duffey et al, 1999). These results were consistent with studies by others which show that inhibition of activation or deletion of NF-κB/RelA inhibits survival of a variety of normal and neoplastic cells of different tissue origin (Beg and Baltimore, 1996; Van Antwerp et al, 1996; Wang et al, 1996; Wu et al, 1996; Bargou et al, 1997; Naksharti et al, 1997; Shattuck-Brandt and Richmond, 1997). These observations indicated that constitutive activation of NF-κB may play a role in inhibiting cell death of HNSCC, even in the absence of TNF-α. Interestingly, independent clones of the UM-SCC-9 cell line in which stable expression of IκBαM was obtained, survived and grew in vitro, but grew poorly or regressed in vivo (Duffey et al, 1999). These observations raise the possibility that even surviving UM-SCC-9 cells transfected with IκBαM may have attenuated resistance to cytotoxic host factors, such as TNF-α.

TNF-α has been reported to have a variety of effects on DNA cell cycle and cell death, including sub G0/G1 DNA fragmentation, and block at the G1/S and G2/M transitions (Watanabe et al, 1987; Coffman et al, 1989; van de Loosdrecht et al, 1993; Wan et al, 1993; Pocsik et al, 1995; Shih and Stutman et al, 1996; Otsuka et al, 1999). The cytotoxic effect of TNF-α on UM-SCC following inhibition of NF-κB or cycloheximide treatment appeared to involve an increase in cell death rather than cell cycle block. The increase in trypan blue staining and sub G0/G1 DNA content of UM-SCC-9 IκBαM transfected cells following TNF-α treatment provides evidence for cell death and subcellular DNA fragmentation. The morphologic changes in UM-SCC-9, -11B and -38 following inhibition of protein synthesis with cycloheximide included cell rounding, blebbing, fragmentation and cell loss (data not shown). The early increase in Sub G0/G1 DNA content in UM-SCC-9I-11 cells and changes in cell morphology of all 3 cell lines following treatment with cycloheximide were observed within 18–24 hours following TNF-α treatment, consistent with the time interval during which TNF-α-induced cell death is observed in other cell types (Beg and Baltimore, 1996; Van Antwerp et al, 1996; Wang et al, 1996).

The susceptibility or resistance of several other cell types to TNF-α induced cell death has recently been shown to depend upon the state of activation or recruitment of signal transduction pathways, particularly those involving transcription factor NF-κB and NF-κB dependent proteins (Beg and Baltimore, 1996; Van Antwerp et al, 1996; Wang et al, 1996). In cells where NF-κB is induced by TNF-α, apoptosis may not occur (Liu et al, 1996). The promotion of cell survival by activation of NF-κB has recently been attributed to expression of several proteins which may protect cells from apoptosis. NF-κB has been reported to induce TRAF1, TRAF2, c-IAP1 and c-IAP2, resulting in suppression of caspase-8 activation, thereby inhibiting apoptosis (Wang et al, 1998). We obtained evidence that TNF-α induces cell death in

 © 2000 Cancer Research Campaign

UM-SCC-9 I11 by a caspase dependent mechanism, since cell death was blocked by a caspase inhibitor. Other novel inhibitors of cell death have been reported. IEX-1L (Wu et al, 1998) and the pro-survival Bcl-2 homologue Bfl-1/A1 (Zong et al, 1999) have been shown to be transcriptional targets of NF-κB which can block TNF-α-induced apoptosis. In other systems, the targets of NF-κB have been shown to include p53 (Hu et al, 1994) and the c-myc oncogene promoter (La Rosa et al, 1994; Klefstrom et al, 1997; Mayo et al, 1997; Bellas and Sourshein, 1999; Kaltschmidt et al, 1999), leading to the abrogation of apoptosis. Our data are consistent with the findings of others which suggest that TNF-α signalling results in a negative feedback mechanism involving NF-κB activation and expression of protective proteins, with subsequent suppression of downstream signals which lead to caspase mediated cell death (Beg and Baltimore, 1996; Van Antwerp et al, 1996; Wang et al, 1996, 1998). Although TNF-α resistance can be inhibited by the addition of cycloheximide in these cell lines, and cytoprotection appears to require new protein synthesis, the identity of these protein(s) in HNSCC remains to be determined.

It is possible that the cytokines expressed by HNSCC also contribute to survival of cells exposed to TNF-α. We previously showed that HNSCC cells express IL-1α (Chen et al, 1998), another cytokine that can induce activation of NF-κB and cytokines (Wood and Richmond, 1995). IL-1α has been reported to promote resistance of cells to apoptosis, such as occurs in response to radiation damage (Neta, 1997). We have recently found that IL-1α serves as an autocrine factor for HNSCC, and that IL-1α can stimulate transcriptional activation of both NF-κB and AP-1 (Wolf et al, 1999). Preliminary studies have provided evidence that expression of IL-1-receptor antagonist to block the autocrine effects of IL1α, produces a decrease in cytokine expression and survival by UM-SCC cell lines (Wolf et al, 1999). Further study in this area is warranted.

Identification of the molecular components of pathways activated up- and downstream of NF-κB in HNSCC will be important. Identification of proteins necessary for cell survival following TNF-α treatment may allow for specific targeting and development of therapy to sensitize HNSCC to TNF-α produced by host responses or given exogenously. For example, epidermal growth factor receptor activation has been detected in the majority of HNSCC, and the EGF induced Ras activation can activate NF-κB and AP-1 (Sun and Carpenter, 1998). Ras activation has been shown to suppress p53 independent apoptosis (Mayo et al, 1997). Since approximately 50% of HNSCC appear to retain wild type p53, it will important to determine whether constitutive activation of NF-κB or AP-1 can prevent apoptosis by p53 mediated DNA repair or p53 independent mechanisms involving c-myc that affect cell cycle (La Rosa et al, 1994; Klefstrom et al, 1997; Bellas and Sonenshein, 1999; Kaltschmidt et al, 1999; Kirch et al, 1999). Regulation or manipulation of activation of these transcription factors, such as by pharmacologic inhibitors of NF-κB (Giardina et al, 1999) or by introduction of mutant transcription factor repressors using viral vectors, may hold promise in sensitizing HNSCC and other cancers to TNF-α and other types of cytotoxic therapy.

### ACKNOWLEDGEMENTS

The authors thank Dr Inder Verma for providing the mutant IκBαM, and Drs Hans Schreiber, Keith Brown and James Battey for reading and commenting on the manuscript. Grant sponsor NIDCD; Intramural project number Z01-DC-00016; Howard Hughes Medical Institute Scholars Program

### REFERENCES

Bargou RC, Emmerich F, Krappmann D, Bommert K, Mapara MY, Arnold W, Royer HD, Grinstein E, Greiner A, Scheidereit C and Dorken B (1997) Constitutive nuclear factor-κB-Re1A activation is required for proliferation and survival of Hodgkin's disease tumor cells. *J Clin Invest* **100**: 2961–2969

Beg AA and Baltimore D (1996) An essential role for NF-κB in preventing TNF-α-induced cell death. *Science* **274**: 782–784

Bellas RE and Sonenshein GE (1999) Nuclear factor kappaB cooperates with c-Myc in promoting murine hepatocyte survival in a manner independent of p53 tumor suppressor function. *Cell Growth Differ* **10**: 287–294

Briskin KB, Fady C, Wang M and Lichtenstein A (1996) Apoptotic inhibition of head and neck squamous cell carcinoma cells by tumor necrosis factor alpha. *Arch Otolaryngol Head Neck Surg* **122**: 559–563

Brockman JA, Scherer DC, McKinsey TA, Hall SM, Qi X, Lee WY and Ballard DW (1995) Coupling of a signal response domain in I kappa B alpha to multiple pathways for NF-kappa B activation. *Mol Cell Biol* **15**: 2809–2818

Brown K, Gerstberger S, Carlson L, Franzoso G and Siebenlist U (1995) Control of IκB-alpha proteolysis by site-specific, signal-induced phosphorylation. *Science* **267**: 1485–1488

Carswell EA, Old LJ and Kassel RL et al (1975) An endotoxin-induced serum factor that causes necrosis of tumors. *Proc Natl Acad Sci USA* **72**: 3666–3670

Chen Z, Colon I, Ortiz N, Callister M, Pegram M, Arosarena O, Strome S, Nicholson JC and Van Waes C (1998) Effects of IL-1α, IL-1RA and neutralizing antibody on proinflammatory cytokine expression by human squamous cell carcinoma lines. *Cancer Res* **58**: 3668–3676

Coffman FD, Haviland DL, Green LM and Ware CF (1989) Cytotoxicity by tumor necrosis factor is linked with the cell cycle but does not require DNA synthesis. *Growth Factors* **1**: 357–64

Dealtry GB, Naylor MS and Fiers W et al. (1987) DNA fragmentation and cytotoxicity caused by tumor necrosis factor is enhanced by interferon-γ. *Eur J Immunol* **17**: 689–693

Dong G, Chen Z, Kato T and Van Waes C (1999) The host environment promotes the constitutive activation of nuclear factor-κB and proinflammatory cytokine expression during metastatic tumor progression of murine squamous cell carcinoma. *Cancer Research* **59**: 1–10

Duffey D, Chen Z, Dong G, Ondrey FG, Wolf JS, Brown K, Siebenlist U and Van Waes C (1999) Expression of a dominant-negative mutant inhibitor-κBα of nuclear factor-κB in human head and neck squamous cell carcinoma inhibits survival, proinflammatory cytokine expression, and tumor growth in vivo. *Cancer Res* **59**: 3468–3474

Fraker DL, Alexander HR and Pass HI (1995) Biologic therapy with TNF: Systemic administration and isolation-perfusion. In: *Biologic Therapy of Cancer* (2nd ed.) V. T. Vincent, Jr., S. Hellman, S. A. Rosenberg. pp 329–345, J.B. Lippincott Company, Philadelphia

Fransen L, Ruysschaert MR and Van der Heyden J et al (1986) Recombinant tumor necrosis factor: Species specificity for a variety of human and murine transformed cell lines. *Cell Immunol* **100**: 260–267

Gapany M, Dawson DE, Schriever C, Burgess R, Gipple JR and Riggs CE Jr (1990) Tumor necrosis factor and cytotoxic agents. Effect on squamous carcinoma lines. *Arch Otolaryngol Head Neck Surg* **116**: 436–9

Giardina C, Boulares H and Inan MS (1999) NSAIDs and butyrate sensitize a human colorectal cancer cell line to TNF-alpha and Fas ligation: the role of reactive oxygen species. *Biochim Biophys Acta* **1448**: 425–38

Haranaka K and Satomi N (1981) Cytotoxic activity of tumor necrosis factor (TNF) on human cancer cells in vitro. *Jpn J Exp Med* **51**: 191–194

Hu H and Lozano G (1994) NF-kappa B activation of p53. A potential mechanism for suppressing cell growth in response to stress. *J Biol Chem* **269**: 20067–20074

Kaltschmidt C, Kaltschmidt B, Henkel T, Stockinger H and Baeuerle PA (1995) Selective recognition of the activated form of transcription factor NF-κB by a monoclonal antibody. *Biol Chem Hoppe Seyler* **376**: 9–16

Kaltschmidt G, Kaltschmidt C, Hehner SP, Droge W and Schmitz ML (1999) Repression of NF-kappaB impairs HeLa cell proliferation by functional interference with cell cycle checkpoint regulators. *Oncogene* **18**: 3213–3225

Kirch HC, Flaswinkel S, Rumpf H, Brockmann D and Esche H (1999) Expression of human p53 requires synergistic activation of transcription from the p53 promoter by AP-1, NF-kappaB and Myc/Max. *Oncogene* **18**: 2728–2738

© 2000 Cancer Research Campaign 

Klefstrom J, Arighi E, Littlewood T, Jaattela M, Saksela E, Evan GI and Alitalo K (1997) Induction of TNF-sensitive cellular phenotype by c-Myc involves p53 and impaired NF-kappaB activation. *EMBO J* **16**: 7382–7392

Knerer B, Hulla W, Martineck H, Formanek M, Temmel and A, Kornfehl J (1996) IL-1 and TNF-alpha but no IL-2 expression is found in squamous cell carcinoma of the head and neck by RT-PCR. *Acta Otolaryngol (Stockh)* **116**: 132–136

Kurokawa H, Yamashita M, Yamashita Y, Murata T, Miura K and Kajiyama M (1998) Estimation of tumor necrosis factor-alpha in the diagnosis, the prognosis and the treatment follow-up of oral squamous cell carcinoma. *Fukuoka Igaku Zasshi* **89**: 312–320

La Rosa FA, Pierce JW and Sonenshein GE (1994) Differential regulation of the c-Myc oncogene promoter by the NF-kappa B rel family of transcription factors. *Mol Cell Biol* **14**: 1039–1044

Larrick JW, Wright SC (1990) Cytotoxic mechanism of tumor necrosis factor-α. *FASEB J* **4**: 3215–3223

Laster SM, Wood JG and Gooding LR (1998) Tumor necrosis factor can induce both apoptic and necrotic forms of cell lysis. *J Immunol* **141**: 2629–2634

Liu ZG, Hsu H, Goeddel DV and Karin M (1996) Dissection of TNF receptor 1 effector functions: JNK activation is not linked to apoptosis while NF-kappaB activation prevents cell death. *Cell* **87**: 565–576

Mayo MW, Wang CY, Cogswell PC, Rogers-Graham KS, Lowe SW, Der CJ and Baldwin AS Jr (1997) Requirement of NF-kappaB activation to suppress p53-independent apoptosis induced by oncogenic Ras. *Science* **278**: 1812–1815

Monchimatsu I, Tsukuda M, Furukawa S, Watanabe S, Kubota A and Yanoma S (1993) The sensitivity of head and neck squamous cell carcinomas to tumor necrosis factor-alpha. *Biotherapy* **6**: 239–244

Nakshatri H, Bhat-Nakshatri P, Martin DA, Goulet RJ Jr and Sledge GW, Jr (1997) Constitutive activation of NF-κB during progression of breast cancer to hormone independent growth *Mol Cell Bio* **17**: 3629–3639

Neta R (1997) Modulation with cytokines of radiation injury: suggested mechanisms of action. *Environ Health Perspect* 105 Suppl. **6**: 1463–1465

Olieman AF, Lienard D, Eggermont AM, Kroon BB, Lejeune FJ, Hoekstra HJ and Koops HS (1999) Hyperthermic isolated limb perfusion with tumor necrosis factor alpha, interferon gamma, and melphalan for locally advanced nonmelanoma skin tumors of the extremities: a multicenter study. *Arch Surg* **134**: 303–307

Ondrey F, Dong G, Sunwoo J, Chen Z, Wolf JS, Crowl-Bancroft CV, Mukaida N and Van Waes C (1999) Constitutive activation of transcription factors NF-κB, AP-1 and NF-IL6 in human head and neck squamous cell carcinoma cell lines that express proinflammatory and proangiogenic cytokines. *Mol. Carcinogenesis* **26**: 119–129

Otsuka G, Nagaya T, Saito K, Mizuno M, Yoshida J and Seo H (1999) Inhibition of Nuclear Factor-κB activation confers sensitivity to tumor necrosis factor-α by impairment of cell cycle progression in human glioma cells. *Cancer Res* **59**: 4446–4452

Parks RR, Yan SD and Huang CC (1994) Tumor necrosis factor-alpha production in human head and neck squamous cell carcinoma. *Laryngoscope* **104**: 860–4

Pocsik E, Mihalik R, Penzes M, Loetscher H, Gallati H and Aggarwal BB (1995) Effect of cell cycle on the regulation of the cell surface and secreted forms of type I and type II human tumor necrosis factor receptors. *J Cell Biochem* **59**: 303–316

Sacchi M, Klapan I, Johnson JT and Whiteside TL (1991) Antiproliferative effects of cytokines on squamous cell carcinoma. *Arch Otolaryngol Head Neck Surg* **117**: 321–326

Schmid DS, Tite JP and Ruddle NH (1986) DNA fragmentation: Manifestation of target cell destruction mediated by cytotoxic T-cell lies, lymphotoxin-secreting helper T-cell clones, and cell-free lymphotoxin-containing supernatant. *Proc Natl Acad Sci USA* **83**: 1881–1885

Schuger L, Dixit VM, Carey TE and Varani J (1990) Modulation of squamous carcinoma cell growth, morphology, adhesiveness and extracellular matrix production by interferon-gamma and tumor necrosis factor-alpha. *Pathobiology* **58**: 279–286

Shattuck-Brandt RL and Richmond A (1997) Enhanced degradation of IκBα contributes to endogenous activation of NF-κB in Hs294T melanoma cells *Cancer Res* **57**: 3032–3039

Shih SC, Stutman O (1996) Cell cycle-dependent tumor necrosis factor apoptosis. *Cancer Res* **56**: 1591–1598

Soylu L, Ozcan C, Cetik F, Paydas S, Kiroglu M, Aydogan B, Sargin O, Ozsahinoglu C and Seyrek E (1994) Serum levels of tumor necrosis factor in squamous cell carcinoma of the head and neck. *Am J Otolaryngol* **15**: 281–285

Sugarman BJ, Aggarwal BB and Hass PE et al. (1985) Recombinant human tumor necrosis factor-α: Effects on proliferation of normal and transformed cells in vitro. *Science* **230**: 943–945

Sun L and Carpenter G (1998) Epidermal growth factor activation of NF-kappaB is mediated through IkappaBalpha degradation and intracellular free calcium. *Oncogene* **16**: 2095–2102

Tartaglia LA, Goeddel DV (1992) Two TNF receptors. *Immunol Today* **13**: 151–153

Traenckner EB, Pahl HL, Henkel T, Schmidt KN, Wilk S and Baeuerle PA (1995) Phosphorylation of human I kappa B-alpha on serines 32 and 36 controls I kappa B-alpha proteolysis and NF-kappa B activation in response to diverse stimuli. *EMBO J* **14**: 2876–2883

Urban JL, Schreiber H (1983) Selection of macrophage-resistant progressor tumor variants by the normal host. Requirement for concomitant T cell-mediated immunity. *J Exp Med* **157**: 642–656

Urban JL, Shepard HM, Rothstein JL, Sugarman BJ and Schreiber H (1986) Tumor necrosis factor: a potent effector molecule for tumor cell killing by activated macrophages. *Proc Natl Acad Sci USA* **83**: 5233–5237

Van Antwerp DJ, Martin SJ, Kafri T, Green DR and Verma I (1996) Suppression of TNF-α-induced apoptosis by NF-κB *Science* **274**: 787–789

Van de Loosdrecht A, Ossenkoppele GJ, Beelen RH, Broekhoven MG and Langenhuijsen MM (1993) Cell cycle specific effects of tumor necrosis factor alpha in monocyte mediated leukemic cell death and the role of beta 2-integrins. *Cancer Res* **53**: 4399–407

Verma IM, Stevenson JK, Schwarz EM, Van Antwerp D and Miyamoto S (1995) Rel/NF-kB/IkB family: intimate tales of association and dissociation. *Gene Dev* **9**: 2723–2735

Von Biberstein SE, Spiro JD, Lindquist R and Kreutzer DL (1995) Enhanced tumor cell expression of tumor necrosis factor receptors in head and neck squamous cell carcinoma. *Am J Surg* **170**: 416–422

Wallach D, Varfolomeev EE, Malinin NL, Goltsev YV, Kovalenko AV and Boldin MP (1999) Tumor necrosis factor receptor and Fas signaling mechanisms. *Annu Rev Immunol* **17**: 331–367

Wan JM, Fogt F, Bistrian BR and Istfan NW (1993) Evaluation of antitumor effect of tumor necrosis factor in terms of protein metabolism and cell cycle kinetics. *Am J Physiol* **265**: 365–34

Wang C-Y, Mayo MW and Baldwin AS (1996) TNF and cancer therapy-induced apoptosis: potentiation by inhibition of NF-κB *Science* **274**: 784–787

Wang C-Y, Mayo MW, Korneluk RG, Goeddel DV and Baldwin AS (1998) NF-κB antiapoptosis: induction of TRAF1 and TRAF2 and c-IAP1 and c-IAP2 to suppress caspase-8 activation. *Science (Wash)* **281**: 1680–1683

Watanabe N, Niitsu Y, Yamauchi N, Neda H, Sone H, Maeda M and Urushizaki I (1987) Cell cycle specificity of tumor necrosis factor and its receptor. *Cell Biol Int Rep* **11**: 813–817

Wolf JS, Chen Z, Dong G, Sunwoo JB and Van Waes C (1999) Induction of cell proliferation and IL-8 expression by autocrine expression of IL-1α is associated with activation of NF-κB and AP-1 in head and neck squamous cell carcinoma. *Proc Amer Assoc for Cancer Research* **1177**

Wood LD and Richmond A (1995) Constitutive and cytokine induced expression of the melanoma growth stimulatory activity GRO alpha gene requires both NF-κB and novel constitutive factors. *J Biol. Chem.* **270**: 30619–30626

Wu M, Lee H, Bellas RE, Schauer SL, Arsura M, Katz D, FitzGerald MJ, Rothstein TL, Sherr DH and Sonenshein GE (1996) Inhibition of NF-kappa B/Rel induces apoptosis of murine B cells *EMBO J* **15**: 4682–4690

Wu MX, Ao Z, Prasad KVS, Wu R and Schlossman SF (1998) IEX-IL, an apoptosis inhibitor involved in NF-κB-mediated cell survival. *Science* **281**: 998–1001

Younes F, Quartey EL, Kiguwa S and Partridge M (1996) Expression of TNF and the 55-kDa TNF receptor in epidermis, oral mucosa, lichen planus and squamous cell carcinoma. *Oral Dis* **2**: 25–31

Zong WX, Edelstein LC, Chen C, Bash J and Gelinas C (1999) The prosurvival BC1–2 homolog Bfl/A1 is a direct transcriptional target of NF-kappaB that blocks TNF alpha-induced apoptosis. *Genes Dev* **13**: 382–387

 *© 2000 Cancer Research Campaign*
